# Impact of remote collaboration-based family physical activity on activity levels and quality of life in children with intellectual developmental disabilities

**DOI:** 10.3389/fpubh.2024.1464099

**Published:** 2024-11-20

**Authors:** Xin Shen, Peiying Huang, Maolin Su, Zijuan Liu, Qian Liu, Yin Guo, Lan Zheng

**Affiliations:** ^1^College of Physical Education, Hunan Normal University, Changsha, China; ^2^Changsha Special Education School, Changsha, China; ^3^Key Laboratory of Physical Fitness and Exercise Rehabilitation of Hunan Province, College of Physical Education, Hunan Normal University, Changsha, China

**Keywords:** students, intellectual and developmental disabilities, moderate to vigorous physical activity, quality of life, family physical activity

## Abstract

**Background:**

Low levels of physical activity (PA) are prevalent among children with intellectual and developmental disabilities (IDD). Implementing family-based physical activities as extracurricular interventions offers a promising approach to enhancing their PA levels and promoting overall health.

**Purpose:**

This study aims to explore a novel integrative strategy by combining family-based activities with school physical education classes, with the objective of enhancing PA levels and improving the quality of life (QoL) for children with IDD. Methods: A total of 36 children with IDD (mean age = 16.4 years) were randomly assigned to a 6-month intervention group (IG) or a control group (CG). Both groups received adjusted adaptive physical education, while the IG received additional family support. Assessments of PA, QoL, and the Physical Activity Enjoyment Scale (PACES) were conducted at baseline, after 6 months, and at a 2-month follow-up post-intervention.

**Results:**

The 6-month intervention results showed that the IG had a significant increase in moderate to vigorous physical activity (MVPA) compared to the CG (*p* < 0.001, *d* = 3.87) and a reduction in sedentary behavior (*p* < 0.001, *d* = 2.28). Additionally, there were improvements in WHOQOL-DIS scores (*p* < 0.001, *d* = 1.61) and PACES scores (*p* < 0.001, *d* = 1.14). At the 2-month follow-up, the IG also showed significant improvements in MVPA, sedentary behavior, and PACES scores, all with *p*-values below 0.001, while no significant change was observed in WHOQOL-DIS scores (*p* = 0.914).

**Conclusion:**

Family-based physical activities facilitated through remote collaboration not only improved the PA levels of children with IDD and enhanced their quality of life, but also positively contributed to the maintenance of long-term healthy behaviors.

## 1 Introduction

Intellectual and developmental disabilities (IDD), which affect approximately 1–3% of the general population (1), are characterized by arrested or incomplete development of cognitive, language, motor, and social abilities during the developmental period (2). Individuals with IDD engage in significantly less physical activity (PA) compared to their non-disabled peers, raising serious health concerns (3, 4). Obesity and overweight rates are notably among students with IDD, which can lead to decreased cardiovascular function, disruptions in glucose and lipid metabolism, and increased susceptibility to various chronic diseases (5–8), ultimately impacting overall health into adulthood. Furthermore, limited PA has been associated with poorer mental health outcomes, including higher levels of anxiety and depression (9, 10).

The World Health Organization (WHO) recommended that engaging in 150–300 min of moderate-intensity or 75–150 min of vigorous-intensity PA weekly can effectively prevent cardiovascular diseases and enhance the quality of life (QoL) for individuals with IDD (11). However, students with IDD tend to show a progressive decline in PA and an increase in sedentary behavior as they age, compared to their neurotypical peers (12, 13). This trend may be attributed to various barriers to PA participation, including limited access to sports facilities, insufficient knowledge about available activities, the unique nature of their disabilities, and a lack of support from families and communities (14–16). This unique context necessitates more structured interventions that emphasize motivational support. While school-based interventions for PA are frequently employed in research to improve PA levels and health outcomes, concerns have been raised regarding their effectiveness in achieving meaningful changes in overall PA levels (17, 18). Addressing these clinical conditions requires more than just a school-based approach; the development of healthy behaviors also depends on ongoing parental support and a conducive extracurricular environment.

Parents, motivated by their commitment to their children’s well-being, play a crucial role in providing essential daily support that facilitates sustained participation in positive health behaviors (19). Research indicates that among children with IDD, greater parental support is significantly associated with increased levels of PA reported by parents or caregivers (20, 21). Such support not only shapes children’s PA behaviors but also cultivates a home environment that encourages engagement in PA (22). However, studies exploring the impact of family support on improving PA and subsequent quality of life for children with IDD remain limited. This gap may stem from the challenges associated with implementing effective interventions. Parents often lack the specialized knowledge and strategies needed to address the unique behavioral changes of children with IDD, which can hinder the sustainability of these interventions. Nevertheless, advancements in technology have made it possible to deliver many traditionally in-person services through remote video consultations (23). This shift presents new opportunities to enhance support and engagement in these interventions.

Therefore, the aim of this study is to explore a novel remote collaboration-based family PA intervention to assess its effectiveness in improving PA levels and QoL among children with IDD. Based on this premise, the study proposes the following hypotheses: (1) Remote collaboration-based family PA will effectively enhance PA and QoL in children with IDD; (2) This program will positively influence the maintenance of long-term healthy behaviors among children with IDD. (3) Relying solely on school-based physical activities provides limited benefits for children.

## 2 Methods

### 2.1 Study design

This 6-month trial was conducted from September 2023 to February 2024 and included three assessment points: pre-intervention, 6 months post-intervention, and 2 months after the intervention concluded. The first 2 weeks served as an adaptation period to enhance comfort and confidence among participants with IDD (24). To determine the necessary sample size for statistical significance, a significance level of 0.05 and an 80% power requirement indicated that at least 14 participants per group were needed. Considering a potential dropout rate of 10% among students with IDD, a minimum of 32 participants was required to ensure reliable results. This randomized controlled trial (Clinical Registration: NCT06444659) was approved by the Ethics Committee of Hunan Normal University (Approval No. 301, 08/05/2023) and conducted in accordance with the Declaration of Helsinki.

### 2.2 Participants

Forty participants were recruited from a specialized school in Changsha, with recruitment and eligibility screening conducted by the school’s physical education teachers. Inclusion criteria consisted of: (1) a diagnosis of mild to moderate intellectual disability (ID) (IQ range: 35–69); (2) age between 14 and 17 years; and (3) at least one family member capable of effective communication with the researchers. Exclusion criteria included: (1) the presence of severe physical disabilities or medical conditions that contraindicate PA, such as severe cardiovascular disease; (2) participation in other exercise programs within the preceding 3 months to mitigate prior PA exposure that could confound the results; and (3) missing more than three intervention sessions to ensure the consistency and reliability of the intervention outcomes.

### 2.3 Intervention

**Figure 1:**
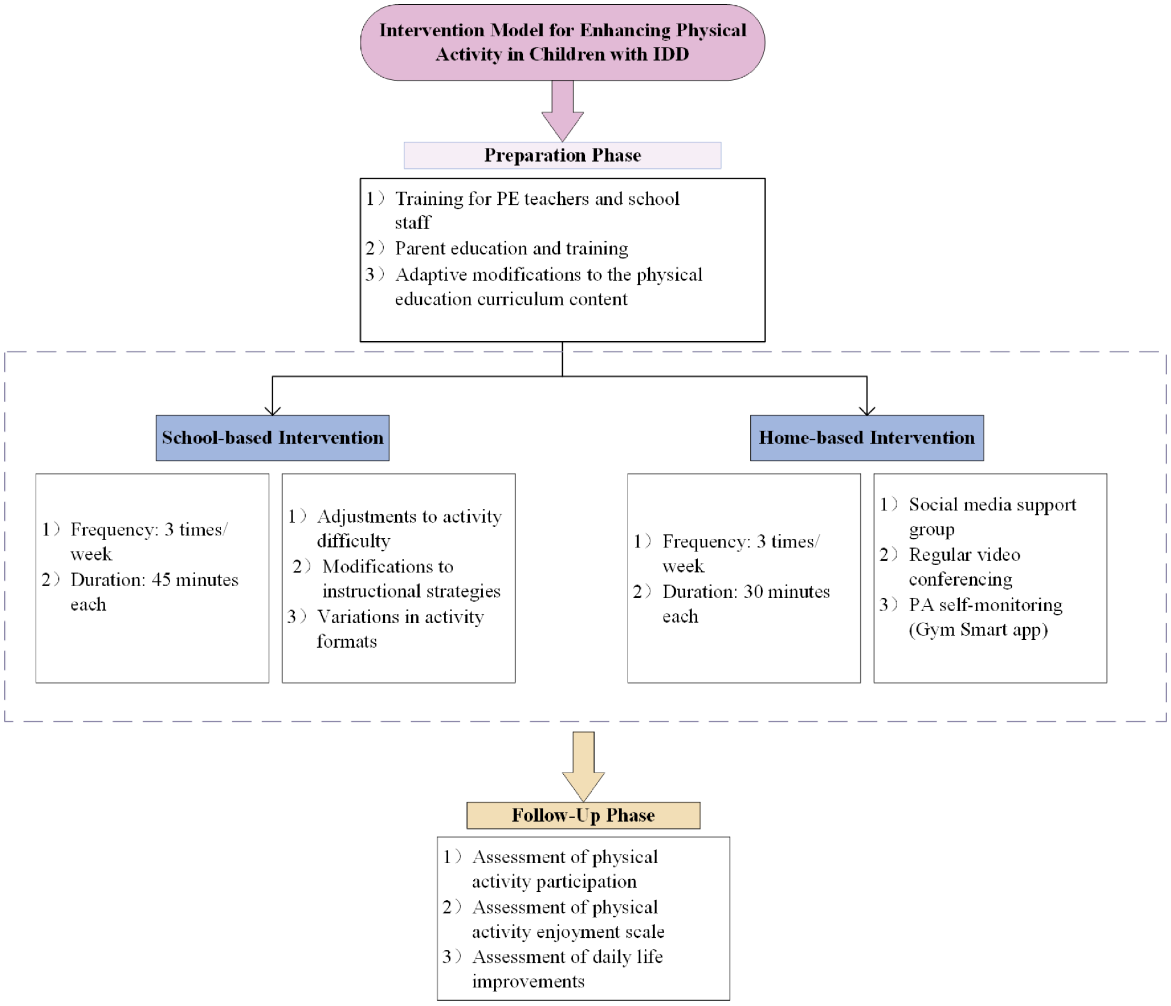
Model of the physical activity intervention.

According to Figure 1, both the intervention and control groups (CG) participated in physical education classes three times per week. To ensure consistency and further investigate the impact of the school-based exercise intervention on the PA levels of children with IDD, the curriculum content underwent specific adaptations. These adaptations included lowering the difficulty level, enhancing teaching strategies through clear language, goal orientation, and visual support, as well as optimizing the instructional content by incorporating games and team activities. These changes were generally considered effective in promoting participation among individuals with IDD (25, 26). Participants in the CG did not receive additional support from the research team, while those in the Intervention Group (IG) received regular remote support as follows:

Before the intervention, all parents received comprehensive PA education and training from experts in education, sports, and health to effectively support their children. Additionally, remote collaborative family PA sessions were conducted via Tencent’s VooV Meeting platform, comprising three 30-min meetings each week. The team consisted of a special education expert and a professor with experience in promoting PA among children. Due to the varying circumstances of each family, the curriculum content was not strictly prescribed; instead, activities were selected based on what children with IDD could accomplish and were willing to engage in, such as various ball games, jump rope, and jogging. Parents utilized video check-ins to ensure the proper implementation of personalized routines, while the Gym Smart (V 1.0; Gezhi Technology, Chengdu, China) was employed to monitor exercise intensity in real time, ensuring safety and facilitating necessary adjustments.

### 2.4 Measures

To evaluate baseline and 6-month PA levels in individuals with IDD, we employed ActiGraph GT3X+ (ActiGraph LLC, Pensacola, FL) triaxial accelerometers. The effectiveness of ActiGraphs in measuring PA levels among IDD students was validated (27, 28). Prior to testing, parents and teachers received training on the proper placement of the accelerometer on the right hip joint (29). IDD students wore the accelerometer continuously for 7 days (5 weekdays and 2 weekends) (30), Removing it only during bathing and sleeping, researchers contacted parents daily to ensure compliance and inquired about accelerometer wear. Real-time supervision occurred during school hours. We applied Choi’s criteria for accelerometer valid data selection to calculate wearing and non-wearing times (31). In line with Chinese adolescent and children’s PA recommendations (32), the accelerometer sampling interval was set at 1 s with a frequency of 60 Hz. Light physical activity (LPA) was defined as 100 to 2,799 counts per minute (CPM), while moderate-to-vigorous physical activity (MVPA) was classified as activity intensity exceeding 2,800 CPM. Assessments were conducted before the intervention, after the intervention, and at a 2-month follow-up following the trial’s conclusion.

Considering the subjective experiences of children with IDD, the WHOQOL-DIS-ID scale was selected to assess their QoL (33). Numerous studies have established its validity and reliability (34, 35). Participants responded to 12 items, each offering five possible answers.

The Physical Activity Enjoyment Scale (PACES) (36) is used to assess enjoyment of PA and has been proven to be reliable and valid. The questionnaire consists of 16 questions rated on a 5-point scale. The scale consists of 9 positively worded items and 7 negatively worded items. Scores for the 7 negatively worded items are reverse-coded and then combined with the scores for the positively worded items. Higher total scores indicate greater levels of enjoyment.

### 2.5 Statistical analyses

All statistical analyses were performed using SPSS version 23.0. Data are presented as mean ± standard deviation (M ± SD). The Shapiro–Wilk test was used to assess the normality of the data distribution. For normally distributed data, paired sample *t*-tests were conducted to evaluate within-group changes. For non-normally distributed data, the Wilcoxon signed-rank test was employed. Accelerometer data, including sedentary time and MVPA, as well as QoL scores, were analyzed using repeated measures ANOVA. Post-hoc analyses were conducted in instances of significant findings. Greenhouse–Geisser correction was applied when the assumption of sphericity was violated.

## 3 Results

### 3.1 Participant characteristics

**Figure 2:**
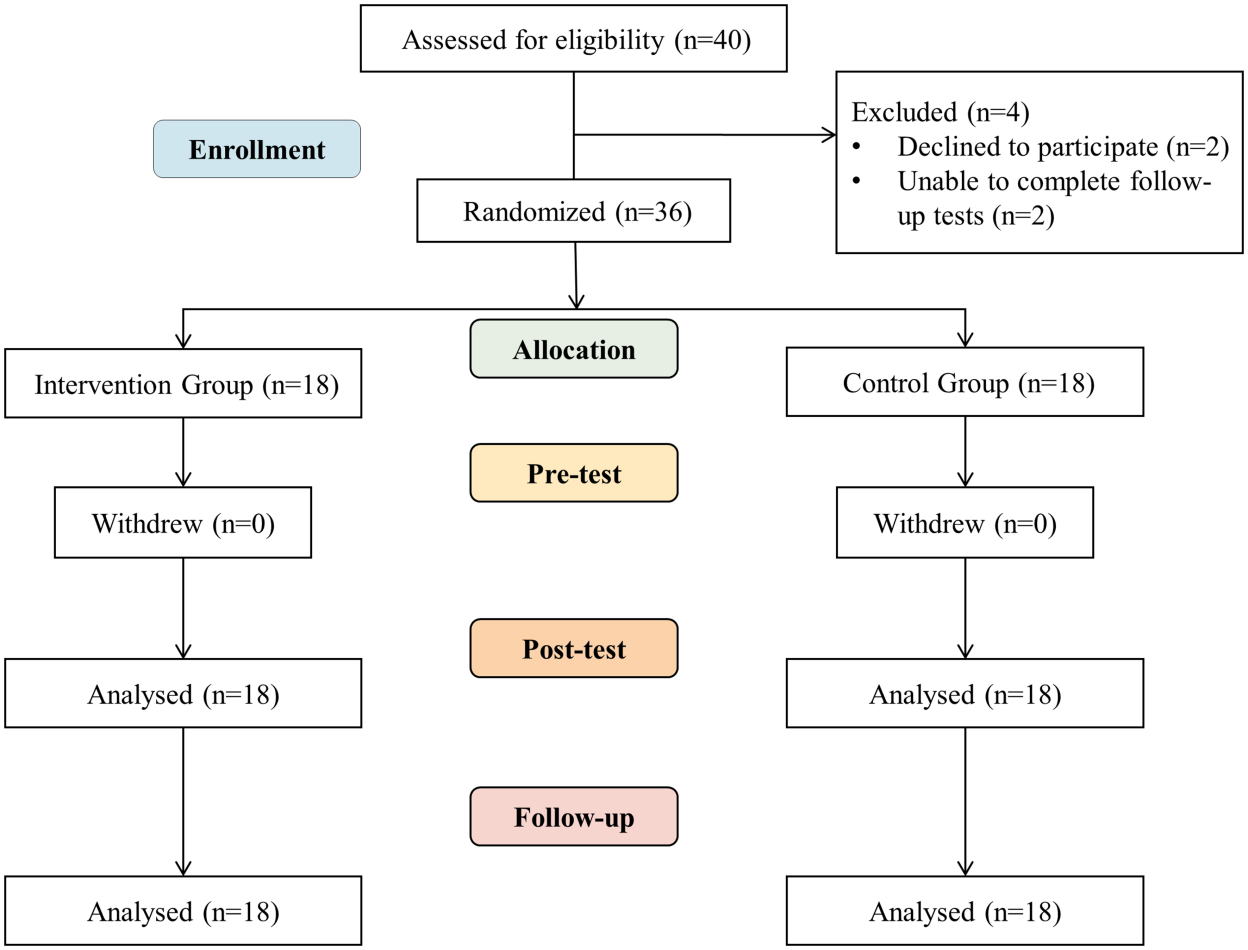
Study flow chart.

As shown in the flow chart (Figure 2), among the 40 participants, 4 withdrew during the intervention: 2 due to lack of effective support from guardians for family reasons, and 2 in the later stages of the trial due to an inability to continue with follow-up tests after contracting influenza.

A total of 36 participants completed the trial, with 58.3% being male and 41.7% female, and an average age of 16.44 years (± 0.73). Among these participants, 23 were diagnosed with mild intellectual disabilities, while 13 were classified with moderate intellectual disabilities. The cohort also included four students with Down syndrome and three students on the autism spectrum. Additionally, one participant designated their sister as the primary contact for the study due to parental circumstances (see Table 1).

**Table 1:** Descriptive characteristics of participants.

Variables	Control group (*n* = 18)	Intervention group (*n* = 18)	Total (*N* = 36)
Age (years)	16.33 ± 0.77	16.56 ± 0.70	16.44 ± 0.73
Height (cm)	166.20 ± 8.37	166.48 ± 7.84	166.34 ± 8.00
Weight (kg)	66.03 ± 9.44	62.63 ± 9.25	64.33 ± 9.37
IQ	51.50 ± 5.75	53.43 ± 7.58	52.46 ± 6.67
BMI	24.04 ± 2.98	22.72 ± 3.57	23.36 ± 3.41
	*n* (%)	*n* (%)	*n* (%)
Gender			
Boys	9 (50%)	12 (66.7%)	21 (58.3%)
Girls	9 (50%)	6 (33.3%)	15 (42.7%)
ID			
Mild (IQ:55–69)	11 (61.1%)	12 (66.7%)	23 (63.9%)
Moderate (IQ:35–54)	7 (38.9%)	6 (33.3%)	13 (36.1%)
Comorbidities			
Down syndrome	2 (11.1%)	2 (11.1%)	4 (11.1%)
Autism	1 (5.6%)	2 (11.1%)	3 (8.3%)
Intellectual disability only	15 (83.3%)	14 (77.8%)	29 (80.6%)

### 3.2 Primary outcome

**Figure 3:**
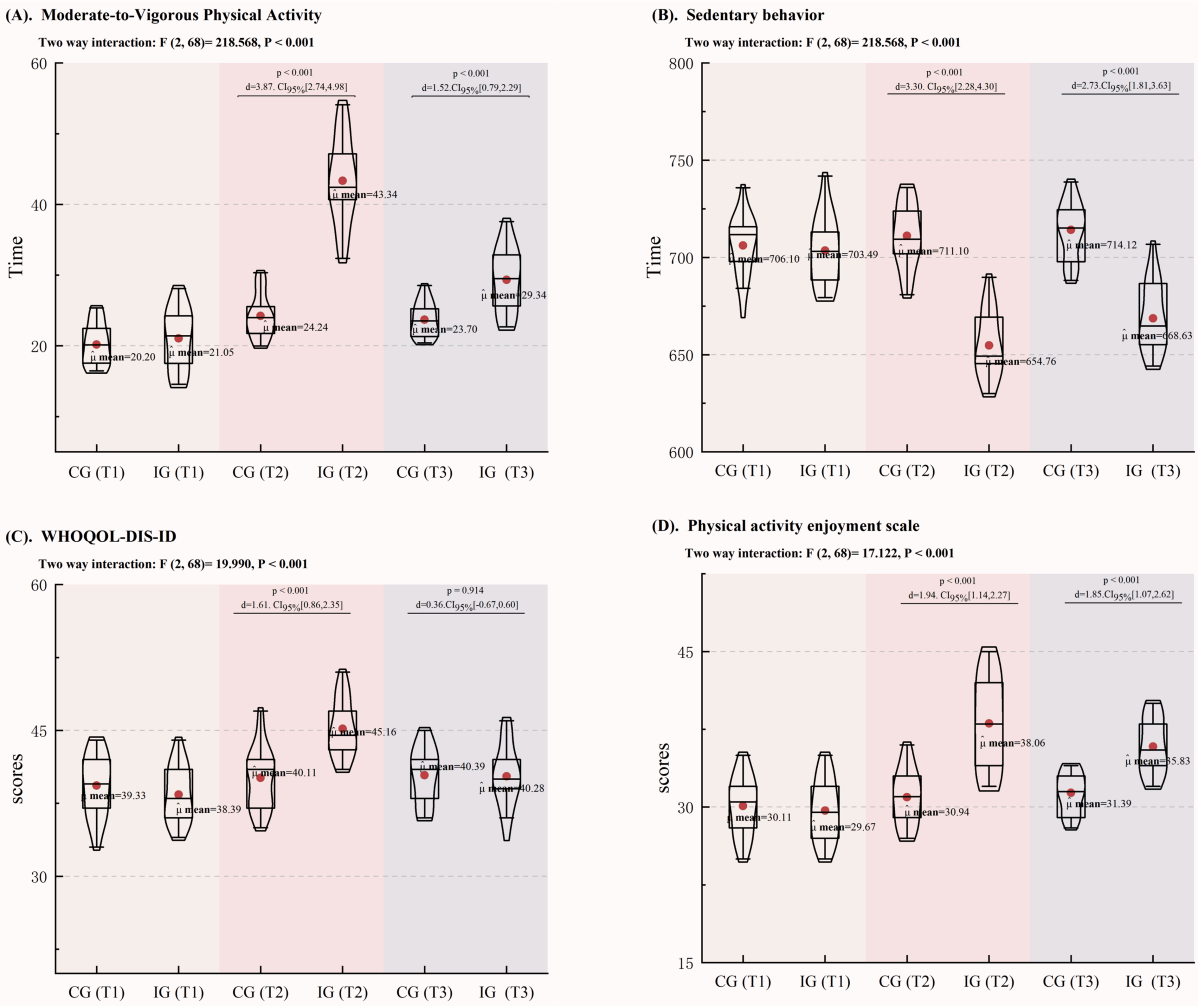
Changes in PA, WHOQoL-DIS, and PACES among Children with IDD. **(A)** Changes in MVPA; **(B)** Changes in Sedentary Behavior; **(C)** Changes in the WHOQoL-DIS-ID; **(D)** Changes in the PACES.

Figure 3 displays the health outcomes of participants in both the IG and CG over a 6-month period, with assessments at T1 (pre-intervention), T2 (6 months post-intervention), and T3 (2-month follow-up). The findings reveal that modifications to the physical education curriculum in the CG did not result in meaningful changes in MVPA, sedentary behavior, QoL scores, or PACES assessments. Conversely, the IG exhibited a noteworthy increase of 19.1 min in MVPA and a reduction of 56.34 min in sedentary time following the intervention. Furthermore, improvements were noted in QoL, with WHOQOL-DIS-ID scores rising by 5.05, and PACES scores increasing by 7.12, all with *p*-values below 0.001.

At the 2-month follow-up (T3), while some metrics in the IG showed a decline, participants still engaged in 5.64 more minutes of MVPA and reduced sedentary behavior by 15.49 min compared to the CG. Additionally, PACES scores remained 4.44 points higher in the IG, with *p*-values consistently below 0.001. These results suggest that the intervention may promote the development of long-term healthy behaviors among children with IDD, despite no significant change in WHOQOL-DIS-ID scores (*p* = 0.914).

## 4 Discussion

Numerous studies have established the pivotal role of parental support in fostering the healthy development of children with IDD (37–39). However, translating this support into practical applications presents significant challenges. This research offers the first empirical evidence highlighting the importance of remote collaborative family activities in sustaining long-term healthy behaviors among children with IDD, thereby enhancing the existing body of literature. Our approach uniquely combines an online platform with a family-oriented PA program, delivering real-time feedback and personalized support tailored to the specific needs of each child. This innovative integration not only encourages active parental engagement, thereby strengthening their role in promoting their children’s health behaviors, but also effectively mitigates barriers that traditional school-based methods may overlook. In comparison to broader school interventions, this model provides a more comprehensive and adaptable strategy for maintaining behavioral change, emphasizing the crucial involvement of families in supporting the health and well-being of children.

Regular participation in physical activity offers numerous lifelong benefits. However, children with IDD face multiple barriers that hinder their engagement and maintenance of physical activity (40, 41), with the role of parents being particularly significant (38, 42). Common obstacles include a lack of parental support, excessive vigilance and protection, and insufficient knowledge about physical activity (43, 44). Addressing these barriers should be a primary focus in promoting physical activity among children with IDD. Parents play an essential role by creating a supportive activity environment, providing emotional encouragement, and instilling healthy values (14, 43). Our findings provide empirical support for this perspective and offer new insights for designing physical activity programs that support parents. Specifically, a family-based activity model facilitated through remote collaboration effectively addresses these challenges, presenting new opportunities for enhancing the health behaviors of children with IDD.

Multiple studies have demonstrated that structured school-based PA positively impacts the health of children with IDD, such as Wang’s 12-week school-based physical intervention, which effectively improved obesity and health-related physical fitness (HRPF) in children with IDD (45). Despite these benefits, children with IDD face various limitations to participation, raising questions about whether school-based activities alone can enhance their PA levels and foster long-term healthy behaviors (18, 52). Our findings support this concern, as the control group showed no significant improvement in physical activity levels, even with some adaptations to the physical education curriculum. When promoting physical activity for children with IDD, it is essential to consider individual factors such as physical skills, cognitive abilities, and self-efficacy, alongside interpersonal influences from teachers, peers, and parents, as well as environmental constraints like community resources and weather conditions (20, 46–48). While existing research underscores the role of physical education classes and recess in facilitating MVPA for children with IDD (49), our results indicate that relying solely on school-based activities may yield limited benefits and fail to sustain long-term healthy behaviors. Therefore, future research should explore the diverse barriers and facilitators influencing participation in physical activity among children with IDD. A focus on parental education and strategies to enhance children’s ongoing engagement will be crucial for fostering sustained behavioral improvements in this population.

The results of the 6-month intervention revealed significant improvements in the PA levels of children with IDD, accompanied by a reduction in sedentary behavior and positive effects on their QoL. Most studies have confirmed that improvements in QoL are closely associated with increased PA (50, 51). This study not only corroborates this relationship but also suggests that this perspective can be extended to the IDD population. The follow-up assessment conducted 2 months later further validated our hypothesis that incorporating family-based physical activities as a supplement to extracurricular activities not only enhances children’s well-being but also fosters the potential for them to maintain active lifestyles and continue engaging in physical activities in the future.

In summary, family-based PA facilitated through remote collaboration offers new insights for promoting the overall well-being of children with IDD. This approach enhances parental involvement in their children’s PA, thereby supporting the maintenance of long-term healthy behaviors. Furthermore, we anticipate that the application of this method will extend beyond improving the QoL for individuals with IDD, potentially benefiting other disability groups that require personalized exercise interventions.

## 5 Study limitations

While this study provides valuable insights, it is important to acknowledge several limitations. First, the relatively small sample size may restrict the generalizability and applicability of the findings. Second, the potential variability in family engagement is a significant limitation, as differences in parental availability, motivation, and understanding of the intervention may affect the consistency and effectiveness of participation in the family-based activities. Future research should address these gaps to better inform the development of tailored programs for children with IDD.

## 6 Conclusion

Family-based physical activities facilitated through remote collaboration have proven effective in improving the PA levels and QoL of children with IDD. This model offers new insights into how to develop exercise programs that actively involve parents and provides empirical evidence to support this approach. Furthermore, the findings underscore the challenges associated with relying solely on school-based physical activities to enhance participation among children with IDD, indicating a need for more targeted and motivationally engaging structured activities.

## Data Availability

The raw data supporting the conclusions of this article will be made available by the authors, without undue reservation.
